# YY1 overexpression is associated with poor prognosis and metastasis-free survival in patients suffering osteosarcoma

**DOI:** 10.1186/1471-2407-11-472

**Published:** 2011-11-02

**Authors:** Filomena de Nigris, Licciana Zanella, Francesco Cacciatore, Anna De Chiara, Flavio Fazioli, Gennaro Chiappetta, Gaetano Apice, Teresa Infante, Mario Monaco, Raffaele Rossiello, Gaetano De Rosa, Marco Alberghini, Claudio Napoli

**Affiliations:** 1 Department of General Pathology, Division of Clinical Pathology and U.O.C. Immunohematology, Second University of Naples, 80138 Naples, Italy; 2 Department of Surgical Pathology, Rizzoli Orthopedic Institute, 40136 Bologna, Italy; 3 Salvatore Maugeri Foundation, Telese-Terme, 82037 Benevento, Italy; 4 Division of Pathology National Cancer Institute, Pascale Foundation, 80131 Naples, Italy; 5 Division of Thoracic Surgery, Sarcoma Team National Cancer Institute, Pascale Foundation, 80131 Naples, Italy; 6 SDN Foundation, Institute of Diagnostic and Nuclear Development, via E. Gianturco 113, 80143 Naples, Italy; 7 Department of Human Pathology, Federico II University of Naples, 80131 Naples, Italy; 8 Department of Human Pathology, Second University of Medicine, 80138 Naples, Italy

## Abstract

**Background:**

The polycomb transcription factor Yin Yang 1 (YY1) overexpression can be causally implicated in experimental tumor growth and metastasization. To date, there is no clinical evidence of YY1 involvement in outcome of patients with osteosarcoma. Prognosis of osteosarcoma is still severe and only few patients survive beyond five years. We performed a prospective immunohistochemistry analysis to correlate YY1 immunostaining with metastatic development and survival in a selected homogeneous group of patients with osteosarcoma.

**Methods:**

We studied 41 patients suffering from osteosarcoma (stage II-IVa). Multivariate analysis was performed using Cox proportional hazard regression to evaluate the correlation between YY1 expression and both metastasis development and mortality.

**Results:**

YY1 protein is not usually present in normal bone; in contrast, a high number of patients (61%) showed a high score of YY1 positive cells (51-100%) and 39% had a low score (10-50% positive cells). No statistical difference was found in histology, anatomic sites, or response to chemotherapy between the two degrees of YY1 expression. Cox regression analysis demonstrated that the highest score of YY1 expression was predictive of both low metastasis-free survival (HR = 4.690, 95%CI = 1.079-20.396; p = 0.039) and poor overall survival (HR = 8.353, 95%CI = 1.863-37.451 p = 0.006) regardless of the effects of covariates such as age, gender, histology and chemonecrosis.

**Conclusion:**

Overexpression of YY1 in primary site of osteosarcoma is associated with the occurrence of metastasis and poor clinical outcome.

## Background

Osteosarcoma is the most common primary malignant bone tumor in adolescents and children [1]. It occurs frequently in long bones and metastasizes preferentially to the lung [1]. Despite recent advances in chemotherapy, the 5-year event-free survival and overall survival rates, closely linked to grade of osteosarcoma, are around 50-60%. This is due to the development of resistance to multiple types of chemotherapy and radiotherapy [2-4]. Clinical stage of the disease and several clinical biomarkers have been correlated with the outcome [5-11]. Nonetheless, these prognostic factors have limited utility in terms of predicting survival [12].

The ubiquitous, conserved, multifunctional polycomb transcription factor Yin Yang 1 (YY1) plays a pivotal role in biological processes [13-15]. YY1 regulates embryonic blood formation and its downstream hox genes, X chromosome inactivation, differentiation, and cell cycle [13,14]. The majority of the data are consistent with the hypothesis that YY1 overexpression and/or its activation is associated with unchecked cellular proliferation, resistance to apoptotic stimuli, tumorigenesis and metastatic potential. We studied the role of YY1 in osteosarcoma carcinogenesis and tumor progression. YY1 is overexpressed in osteosarcoma cells and bioptic specimens, and is correlated with a high degree of malignancy [16,17]. Moreover, YY1 silencing has been shown to be sufficient to significantly reduce osteosarcoma metastatic growth and neoangiogenesis in a nude mice model [18-20]. To date, there is no evidence of correlation between YY1 overexpression and clinical outcome in osteosarcoma patients. Thus, we designed a prospective study to analyze whether YY1 expression in the primary tumor of osteosarcoma patients could predict metastasis-free and overall survival.

## Methods

### Patients

We enrolled 41 osteosarcoma patients (stage II-IVa UICC/AJCC classification) from the Department of Pathology of the Istituto Ortopedico Rizzoli (Bologna, Italy) and from the Division of Surgical Pathology, Istituto Nationale Tumori, Fondazione G. Pascale (Naples, Italy), under their Local Ethical Committee approval. We used the bioptic samples of primary tumor before any treatment (see below). Of the 41 patients, 14 had metastasis at the first visit (synchronous), 15 developed metastasis during follow-up (metachronous) and 12 were metastasis-free. Metastases were localized in lung and the primary sites were in extremity bones. Extraskeletal, periosteal, and paraosteal osteosarcomas were excluded from this study. All slides of the cases were reviewed by two pathologists to confirm diagnosis. Patients received preoperative, postoperative or no chemotherapy according to degree of tumor stage. Necrosis area was defined by using the Huvos grading system, as described in detail [21,22]. Accordingly, we subdivided patients into two groups (<90%) and (≥90%) based on chemonecrosis area as indicated by the European Cooperative Osteosarcoma Study Group coordinated by the Istituto Ortopedico Rizzoli (COSS) [21,22], a partner of the present study.

Chemotherapy protocols included methotrexate (12 g/m^2^) with leucovorin rescue, cisplatin (90-150 mg/m^2^), doxorubicin (60-90 mg/m^2^), and ifosfamide (6-10 g/m^2^). The scheduled duration of chemotherapy ranged from 24 to 38 weeks. For chemotherapy patients, surgery was scheduled to take place between weeks 9 and 11 and radiotherapy was not used. We collected clinical data from all patients including age, sex, tumor site, necrosis area after chemotherapy and surgical stage.

### Immunohistochemistry

Biopsies before chemotherapy were fixed and paraffin embedded. Conventional immunohistochemical studies were performed on 5-6 μm section, as previously described in detail [16,22]. Briefly, slides were immersed in a water bath (W-cap Bioptica) and incubated for 30 minutes at 95°C, then cooled for 20 minutes at room temperature. Sections were then incubated for 10 minutes in 3% hydrogen peroxide in distilled water, and washed in PBS for 5 minutes. Slides were incubated with the primary antibody (mouse-monoclonal YY1 diluted 1:100 sc7341 Santa Cruz) for 30 minutes in a Dako Autostainer device. The signal was then visualized with streptavidina-biotina Dako REAL Detection System Peroxidase/DAB, Rabbit/Mouse Dako Autostainer. Slides were counterstained with hematoxylin. Washing steps were included after each incubation. All samples were stained with vimentin as tissue quality controls while positive and negative conventional controls were used to test antibody as already reported [16]. All controls gave satisfactory and reproducible results. Digital photos were obtained with Olympus Vanox AHBS3. The staining of tumor cells for evaluation of YY1 was scored from 0 to 4 (0=no staining; 1=weak 1-25% positive cells; 2= moderate 26%-50% positive cells; 3= strong 51%-75% positive cells; 4= very strong 76%-100% positive cells. This score was checked by two independent observers blinded for clinical outcome or other biological tumor features. Discrepant cases were decided on consensus.

### Statistical analysis

Data were analyzed with SPSS 13.0 software. Results are reported as mean ± SD. YY1 was considered as a discrete variable and the patients were stratified into two classes: high score as 3-4, (>51-100% positive cells) and low score as 0-2, (0-<50% positive cells) of YY1. ANOVA was used to assess difference for continuous variables (age and mean follow-up) while Pearson chi square test was used to examine differences between YY1 expression and each clinical variable: gender, tumor site, histotype, chemonecrosis, presence of metastasis at diagnosis and occurrence of metastasis at follow-up (n = 41). Survival periods were counted in months from the date of first visit to date of death or last follow-up before study closure. Follow-up data started at time of diagnosis allowing a minimum of 3.8 months (mean 47.0 ± 25.3 month range, 3.8-95 months). Mean age of the sample was 19.4 ± 15.5 with a range from 4 to 76 years. Metastasis-free period was counted in months from the date of surgery to date of detection of first metastasis, since no local relapses occurred. We used life-table method to estimate the metastasis-free survival and overall survival for low and high levels of YY1 expression. Multivariate Cox regression analysis was used to evaluate the effect of YY1 expression on metastasis occurrence and survival independently of age, gender, histotype and chemonecrosis. A p value <0.05 was considered as statistically significant.

## Results

### YY1 overexpression in osteosarcoma tissues

**Figure 1:**
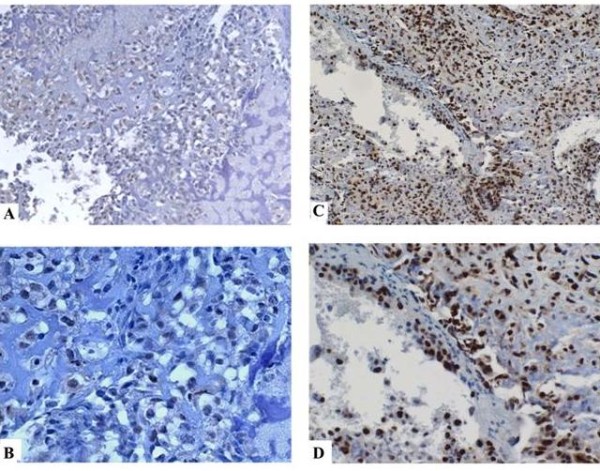
**Two comparative examples of YY1 expression in osteosarcomas**. **A**, osteobastic osteosarcoma specimen with 2 as score of immunoreactivity to YY1 at lower magnification (20x); **B**, the same specimen as in A at higher magnification (100x); **C**, osteoblastic osteosarcoma specimen with 4 as score of immunoreactivity to YY1 (20x); **D**, the same osteoblastic osteosarcoma specimen as in **C **at higher magnification (100x). YY1 protein was localized in nuclei of the cells.

**Figure 2:**
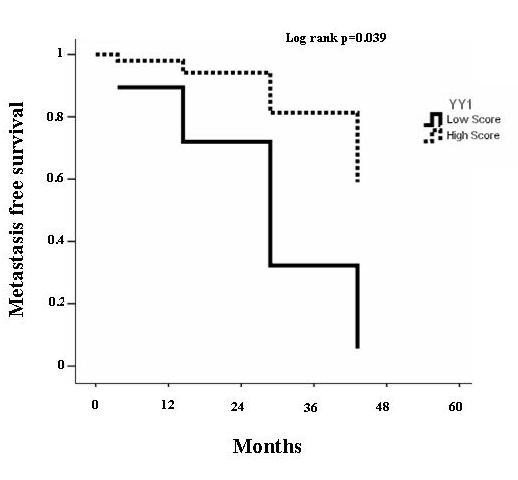
**Cox regression of cumulative metastasis-free survival rate**. The graph shows the cumulative metastasis-free survival of high grade osteosarcoma patients using Cox multivariate analysis. The data indicates that high score of YY1 is associated with higher probability of developing metastasis during the follow up (HR = 4.690, 95%CI = 1.079-20.396; p = 0.039). HR= hazard ratio; CI=confidence interval.

**Figure 3:**
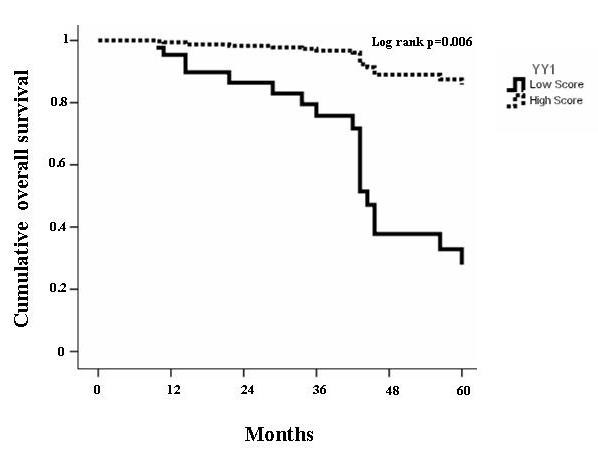
**Cox regression of cumulative survival rate of all patients**. The graph shows the cumulative overall survival rate of high grade osteosarcoma patients (n = 41) using Cox multivariate analysis. The data indicates that higher score of YY1 (3-4) predicts mortality during the follow up. (HR = 8.353, 95%CI = 1.863-37.451, p = 0.006) HR= hazard ratio; CI=confidence interval.

Immunohistochemistry revealed that all normal bone tissues analyzed (n = 10) were almost negative to YY1 antibody or in some cases normal bone showed faint and diffuse cytoplasm staining, as previously reported [16]. Figure 1 shows two representative examples of osteosarcoma with the low score (2) and high score (4) of YY1 antibody localized predominantly in nuclei (95%). Table 1 shows YY1 scoring with respect to clinical variables. Table 2 summarizes the characteristics of the study population stratified by high and low YY1 expression (see methods). A total of 61% of tumor samples were positive to YY1 staining (nuclear staining) with strong intensity (score 3-4, >51-100% positive cells) while 39% showed a lower degree of YY1 staining (score 1-2, <50% of positive cells). Distal femur and tibia were the most common sites (53.7% and 19.5% respectively). The majority of tumors arose on extremities. There was no difference in degree of YY1 expression in histological subgroups (i.e. chondroblastic, fibroblastic and osteoblastic types, p = 0.557) and age. Twenty-seven patients were free of metastasis at baseline while 15 (55.6%) developed metastasis during follow-up. Of these, 80% were positive to YY1 (51-100% positive cells) (p = 0.004). In univariate analysis, death occurred in 64% of YY1 strong positive patients (p = 0.005). Importantly, multivariate Cox regression analysis revealed that a high level of YY1 expression (score 3-4) was predictive of poor metastasis-free survival (HR = 4.690, 95%CI = 1.079-20.396; p = 0.039) (Table 3 and Figure 2). The estimated overall survival (after 60 months of follow-up) calculated by life-table method (Table 4) was 34% for patients with a high YY1 score and 79% for those with a low YY1 score. Multivariate Cox regression analysis indicated that YY1 expression is predictive of mortality (HR = 8.353, 95%CI = 1.863-37.451, p = 0.006) as an independent variable with respect to age, gender, histotype and chemonecrosis (Figure 3 and Table 5).

**Table 1:** YY1 scoring as continuous variable

	YY1 - 0	YY1 - 1	YY1 - 2	YY1 - 3	YY1 - 4
Clinical features	4 patients	9 patients	4 patients	9 patients	15 patients
Age	16.3 ± 7.4	30.1 ± 23.9	13.0 ± 2.2	12.3 ± 8.9	19.7 ± 13.4
**Gender**					
Male	25.0	66.7	100.0	66.0	73.3
**Chemonecrosis ≥ 90%**	50.0	33.3	25.0	33.3	40.0
Metachronous metastasis (%)*	0	12.5	75.0	60.0	88.9
**Mortality**	25.0	22.2	50.0	66.7	53.3
Mean follow -up	50.3 ± 31.6	39.7 ± 21.0	62.0 ± 37.3	43.6 ± 23.0	48.5 ± 25.6

*Data reported are from the 27 patients free of metastasis at baseline and expressed in percentage

**Table 2:** Univariate analysis of YY1 expression and clinical pathological characteristics of patients

Clinical features	Number of cases 41	YY1(low) (0-50% positive cells) 16 cases (39.0%)	YY1 (strong) (51%-100% positive cells) 25 cases (61.0%)	p
Age (4-76)	19.4 ± 15.6	24.4 ± 19.1	16.2 ± 12.0	0.099
**Gender**				
Male	68.3	62.5	72.0	0.524
**Chemonecrosis ≥ 90%**	36.6	37.5	36.0	0.923
**Synchronous metastasis (%)**	34.1	25.0	40.0	0.323
Metachronous metastasis (%) *	55.6	25.0	80.0	0.004
Mean time to metastasis (range 3-76 months)	29.4 ± 19.2	37.6 ± 22.9	22.9 ± 13.1	0.047
**Mortality**	46.3	18.8	64.0	0.005
Mean follow-up (range 3-95 months)	47.0 ± 25.3	49.8 ± 27.5	45.2 ± 24.2	0.578

* Data reported are from the 27 patients free of metastasis at baseline

**Table 3:** Multivariate Cox regression analysis on incidence of metachronous metastasis

Variable	HR	Metachronous metastasis (95.0% CI)	p
Age	1.005	0.969-1.043	0.772
Gender (male)	1.057	0.230-4.850	0.944
Histology	1.201	0.321-4.493	0.786
Chemonecrosis ≥ 90%	0.717	0.219-2.348	0.582
YY1 in primary site (strong vs low)	4.690	1.079-20.396	0.039

HR= Hazard Rate

**Table 4:** Association between YY1 overexpression and probability of survival and median survival

Years after the first visit	% of survival patients 41 cases	% of survival patients per year stratified for YY1	
		YY1 Low (16 cases)	YY1 strong (25 cases)
1	95	94	96
2	88	94	84
3	77	86	72
4	53	79	39
5	50	79	34
6	50	79	34
7	50	79	34
Median survival	58.4	84	44

**Table 5:** Multivariate Cox regression analysis of clinical variables on overall survival

Variable	HR	(95% CI)	p
Age	1.046	1.014-1.079	0.004
Gender (male)	0.628	0.187-2.110	0.452
Metastasis	1.282	0.462-3.558	0.634
Histology	0.780	0.365-1.666	0.521
Chemonecrosis ≥ 90%	0.293	0.097-0.884	0.029
YY1 in primary site (strong vs low)	8.353	1.863-37.451	0.006

HR = Hazard Rate

## Discussion

The present study demonstrates that a high level of YY1 protein expression increases the risk of metastasis (4.69-fold) and poor survival (8.35-fold) in osteosarcoma patients independently of covariates such as age, gender, histotype, and chemonecrosis. We report that the highest range of YY1 expression is a statistically significant prognostic factor setting the 5-year survival rate to 34% in patients with osteosarcoma. These results are in line with literature data and with the tumor necrosis rate which is currently the strongest clinical prognostic factor after chemotherapy [2,23,24].

Overall, the molecular complexity of osteosarcoma makes the known prognostic markers of limited utility [12,25]. A multiple panel of biomarkers in addition to clinical parameters would be useful for predicting prognosis [25]. In this setting, YY1 is the first osteosarcoma marker whose overexpression has been correlated with low metastasis-free and poor overall survival in a higher frequency of cases (61% in the present study) than reported in other studies leading us to set a higher cut-off value (YY1 > 50%) [12]. In addition, low YY1 expression was correlated with best clinical prognosis and absence of metastasis during follow-up. One common limitation of immunohistochemical studies is both antibody sensitivity and specificity. To address these issues, we used an antibody previously tested by other groups [26,27]. Immunohistochemistry was also performed in two different Institutions which studied different subgroups of patients [26,27]. YY1 was localized in the nucleus irrespective of histologic subtype, patient age, or tumor site. Although this is a small study, there was no significant difference in YY1 scores between younger and older age groups suggesting its role in tumor development. The design of the present study stemmed from our previous in vitro observations demonstrating that YY1 was overexpressed in osteosarcoma cells and tissues with more aggressive phenotype [16-18]. This is in agreement with YY1 overexpression in prostate, gastrointestinal [26,27] and other tumors [14]. Moreover, in an in vivo mice model of osteosarcoma YY1 was also shown to play a key role in metastatic growth [19] by regulating vascular supply [20,28].

Interestingly, the majority of patients analyzed revealed strong YY1 expression and showed poor response to chemotherapy based on Huvos grading system. Although the correlation between YY1 expression and prognosis may merely be a statistical association, the protein dosage points to a functional relationship between YY1 overexpression and a more aggressive tumor phenotype. Noteworthy, literature data from other tumoral and non-tumoral tissues provide at least some evidence for the proposed functional relationship. Resistance to Fas-mediated apoptosis of prostate cancer cells is linked to YY1 [29]. A further potential link between YY1 overexpression and chemoresistance lies in the fact that YY1 regulates DR5 gene [30]. We have recently demonstrated that YY1 is involved in CXCR4 and VEGF pathways [19,20] which have both been genetically amplified and correlated with poor survival of soft-tissue sarcomas [8,31,32]. In addition, YY1 overexpression increases resistance to taxana treatment in epithelial ovarian cancer [32]. Furthermore, in a meta-analysis, YY1 was scored as the most significant gene upregulated in metastatic breast cancer [33]. Thus, YY1 screening may be useful in optimizing individual therapy management at the time of diagnosis in these patients.

In conclusion, we demonstrated for the first time that high YY1 expression in osteosarcoma is associated with metastasis development and mortality independently of age, gender, histotype and presence of metastasis at baseline. While other studies have analyzed larger cohorts of patients [34,35], this is the first time a prognostic marker has demonstrated poor outcome by multivariate analysis. If data are confirmed in a larger cohort of patients, YY1 may become part of a multiple panel of biomarkers clinically useful for osteosarcoma prognosis.

## Conclusions

Our study established that overexpression of YY1 in primary site of osteosarcoma is associated with the occurrence of metastasization and poor clinical prognosis. This may be a novel marker for patients with osteosarcoma.

## Competing interests

All authors have no potential conflict of interest including any financial, personal or other relationships with other people or organizations within that could inappropriately influence (bias) their work.

## Authors' contributions

LZ, RR and GC performed immunohistochemistry experiments. FC performed statistical analysis and had responsibility for integrity of database. AM and DC grading evaluations of osteosarcoma patients. FDN and CN designed the study and wrote the manuscript. All the authors read and approved the final version of the manuscript.

## Pre-publication history

The pre-publication history for this paper can be accessed here:

http://www.biomedcentral.com/1471-2407/11/472/prepub
